# Safety and Efficacy of Fecal Microbiota Transplantation for Grade IV Steroid Refractory GI-GvHD Patients: Interim Results From FMT2017002 Trial

**DOI:** 10.3389/fimmu.2021.678476

**Published:** 2021-06-17

**Authors:** Ye Zhao, Xuewei Li, Yujing Zhou, Jin Gao, Yang Jiao, Baoli Zhu, Depei Wu, Xiaofei Qi

**Affiliations:** ^1^ Department of Hematology, The First Affiliated Hospital of Soochow University, Suzhou, China; ^2^ National Clinical Research Center for Hematologic Diseases, Jiangsu Institute of Hematology, Collaborative Innovation Center of Hematology, Suzhou, China; ^3^ Institute of Blood and Marrow Transplantation, Soochow University, Suzhou, China; ^4^ Key Laboratory of Thrombosis and Hemostasis of Ministry of Health, The First Affiliated Hospital of Soochow University, Suzhou, China; ^5^ Cyrus Tang Hematology Center, Soochow University, Suzhou, China; ^6^ Center for Clinical Laboratory, The First Affiliated Hospital of Soochow University, Suzhou, China; ^7^ State Key Laboratory of Radiation Medicine and Protection, School of Radiation Medicine and Protection, Soochow University, Suzhou, China; ^8^ Chinese Academy of Sciences (CAS) Key Laboratory of Pathogenic Microbiology and Immunology, Institute of Microbiology, Chinese Academy of Sciences, Beijing, China; ^9^ Savaid Medical School, University of Chinese Academy of Sciences, Beijing, China; ^10^ Beijing Key Laboratory of Antimicrobial Resistance and Pathogen Genomics, Institute of Microbiology, Chinese Academy of Sciences, Beijing, China; ^11^ Department of Pathogenic Biology, School of Basic Medical Sciences, Southwest Medical University, Luzhou, China; ^12^ Department of Urology, The First Affiliated Hospital of Soochow University, Suzhou, China

**Keywords:** fecal microbiota transplantations, refractory gastrointestinal, graft-versus-host disease, diarrhea, clinical trials

## Abstract

Gastrointestinal (GI) tract graft-versus-host disease (GvHD) is a major cause of post-allo-HSCT (hematopoietic stem cell transplantation) morbidity and mortality. Patients with steroid-refractory GI-GvHD have a poor prognosis and limited therapeutic options. FMT2017002 trial (#NCT03148743) was a non-randomized, open-label, phase I/II clinical study of FMT for treating patients with grade IV steroid-refractory GI-GvHD. A total of 55 patients with steroid-refractory GI-GvHD were enrolled in this study. Forty-one patients with grade IV steroid-refractory GI-GvHD were included in the final statistical analysis. Of them, 23 patients and 18 patients were assigned to the FMT group and the control group, respectively. On days 14 and 21 after FMT, clinical remission was significantly greater in the FMT group than in the control group. Within a follow-up period of 90 days, the FMT group showed a better overall survival (OS). At the end of the study, the median survival time was >539 days in the FMT group and 107 days in the control group (HR=3.51; 95% CI, 1.21–10.17; *p*=0.021). Both the event-free survival time (EFS) (HR=2.3, 95% CI, 0.99–5.4; *p*=0.08) and OS (HR=4.4, 95% CI, 1.5–13.04; *p*=0.008) were higher in the FMT group during the follow-up period. Overall, the mortality rate was lower in the FMT group (HR=3.97; 95% CI, 1.34–11.75; *p*=0.013). No differences in the occurrence of any other side effects were observed. Our data suggest that the diversity of the intestinal microbiota could be affected by allo-HSCT. Although its effectiveness and safety need further evaluation, FMT may serve as a therapeutic option for grade IV steroid-refractory GI-GvHD.

**Clinical Trial Registration:**

[ClinicalTrials.gov], identifier [NCT03148743].

## Introduction

Hematopoietic stem cell transplantation (HSCT) can be used to treat most cases of acute leukemia; however, HSCT may cause many complications, including infections, multi-organ failure, and graft-versus-host disease (GvHD) (1–3). GvHD, especially gut acute GvHD (GI-aGvHD), is a major cause of post-allo-HSCT morbidity and mortality (3, 4).

Conventionally, glucocorticoids are used as the first-line therapy for GI-GvHD. Unfortunately, almost half of the patients do not respond well to glucocorticoids (3–5). The survival of patients with GI-GvHD treated with standard steroid regimen ranges between 5% and 30% (4–8). Few second-line treatments have been established, and they are urgently needed (5, 8).

The human gut microbiota, which is composed of more than 100 trillion microbes, is associated with many chronic diseases (9). The influence of intestinal microbiota on immune responses, including post-allo-HCT, has been increasingly recognized (10–12) and has become one of the main treatment targets for acute GvHD (3, 8). The diversity of the gut microbiota participates in intestinal inflammation in normal conditions (3, 13). After allo-HSCT, the intestinal microbial diversity collapses (14, 15), which may damage GI mucosa and consequently influence the immune response (3).

Fecal microbiota transplantation (FMT) is a clinical procedure that infuses a fecal suspension from a healthy donor into the recipient’s GI tract. FMT can quickly restore the recipient’s intestinal microbiota, increase regulatory T cells and short-chain fatty acids, repair the intestinal mucosal barrier, which may resolve the inflammatory response and readjust the immune system (16, 17). Moreover, steroids aggravate GI tissues damage and affect GI tissues repair. FMT is beneficial to GI tissue repair (5). Thus, it is reasonable to perform FMT in SR-GvHD patients. As a novel therapeutic method, FMT has been proven to be effective for recurrent *Clostridium difficile* infection (18, 19).

Our pilot study and other published studies suggested that FMT could serve as a therapeutic option for treating steroid-refractory GI-GvHD (3, 14, 15, 20). Here, we assessed the safety and efficacy of FMT in a phase I/II study involving patients with grade IV steroid-refractory gastrointestinal tract GvHD.

## Patients and Methods

### Study Design and Participants

An open-label, non-randomized phase I/II clinical study was conducted at the First Affiliated Hospital of Soochow University. Protocols and other trial-related procedures were approved by the Institutional Review Board of the hospital. Written informed consent was obtained from all the patients. The FMT and control groups (without FMT) were set according to the patients’ decision after introducing the possible benefits and disadvantages of fecal bacteria transplantation. FMT was performed after steroid-refractory GI-GvHD had been diagnosed. All the patients received a second-line immunosuppressant treatment. Only the FMT group received FMT. The Center for International Blood and Marrow Transplant Research (CIBMTR) criteria were used to assess the grades of GI-GvHD (3, 21, 22). The criteria for diagnosing steroid-refractory gut GvHD were described previously (3). Patients with uncontrollable infection, irreversible organ failure, and other abnormal conditions that might interfere with the evaluation were excluded from the study (online supplement protocol).

The study was registered with ClinicalTrials.gov as #NCT03148743.

### FMT Procedures

The fecal materials were handled in sealed, fully automatic machines (GenFMTer, Nanjing, China). The fecal microbiota samples collected from four healthy donors (two women aged 23 years and two men aged 20 years) were conserved at −80°C with glycerine (online supplement protocol). As these patients did not tolerate gastroscopy or enteroscopy, 40–50 mL of frozen fecal microbiota was suspended in 150–200 mL of warm normal saline and delivered into the intestine of the recipients through a nasojejunal or gastric tube after diagnosing grade IV steroid-refractory GI-GvHD (3). If there was no improvement, FMT was repeated in the following week.

### Outcomes

The primary outcomes were event-free survival time (EFS) and overall survival (OS) on day 90 after the diagnosis of steroid-refractory GI-GvHD; EFS and OS after steroid-refractory GI-GvHD were recorded until November 1, 2018.

Secondary outcomes were clinical remission or partial remission on days 14, 21, and 28 after the diagnosis of steroid-refractory GI-GvHD.

The efficacy of FMT was evaluated according to the severity of symptoms, such as abdominal pain, diarrhea (frequency and volume), and bloody purulent stool within 14 and 21 days after FMT. The abdominal pain scores were assigned as follows: occasional pain (0.5), mild pain (1), moderate pain (2), severe pain without intervention (3), and severe pain (4). Clinical remission was defined as a condition in which diarrhea and intestinal spasms and/or bleeding disappeared or stool volume decreased by ≥500 mL on average within 3 days. Clinical improvement was defined as a condition in which the stool volume decreased by <500 mL, or the abdominal pain value and bleeding were relieved. EFS was defined as the period during the follow-up after the first FMT with no progression of GI-GvHD, no death, no GvHD involvement of other organs, and no new infection with cytomegalovirus (CMV) or Epstein–Barr virus (EBV) (3). OS referred to the period from the diagnosis of steroid-refractory GI-GvHD to November 1, 2018. All deaths in this period, including those related to relapse and those due to other causes, were included in the statistical analysis.

For each patient, safety was evaluated according to adverse events (including death or drop-out) during FMT and the follow-up period.

### Stool Sample Collection and Microbial Community Analysis

Fecal samples were stored at −80°C until DNA extraction. After DNA extraction, bacterial 16S rDNA was successfully detected in all of the samples by polymerase chain reaction (PCR) using general bacterial primers (16S V4-V5): 515F:5’-GTGCCAGCMGCCGCGGTAA-3’; 926R: 5’-CCGTCAATTCMTTTGA -GTTT-3’. After purification, the pooled libraries were sequenced by 2×300 bp paired-end sequencing on the MiSeq platform (Tiny Gene, Shanghai, China) using the MiSeq v3 Reagent Kit (Illumina). Mothur, UPARSE, and R software were used to analyze the 16S sequencing data. The composition of fecal bacteria was analyzed at the phylum level. Moreover, the Shannon diversity index was used to depict the diversity of the microbiota (online supplement methods) (3, 23).

### Statistical Analysis

The statistical software SPSS 16.0 (SPSS, Inc., IL, USA) was used to construct actuarial rate curves, to calculate log-rank hazard ratios (HRs) and Fisher’s exact tests, and to perform significance and risk determinations. Cochran’s and Mandel-Haenszel statistical methods were used to examine the differences between the groups. The ‘survival’ package in R statistical software (Vienna, Austria) was used for the permutation tests. Significance was determined using Cox proportional-hazards models with time-varying covariates.

## Results

### Patient Characteristics

**Figure 1:**
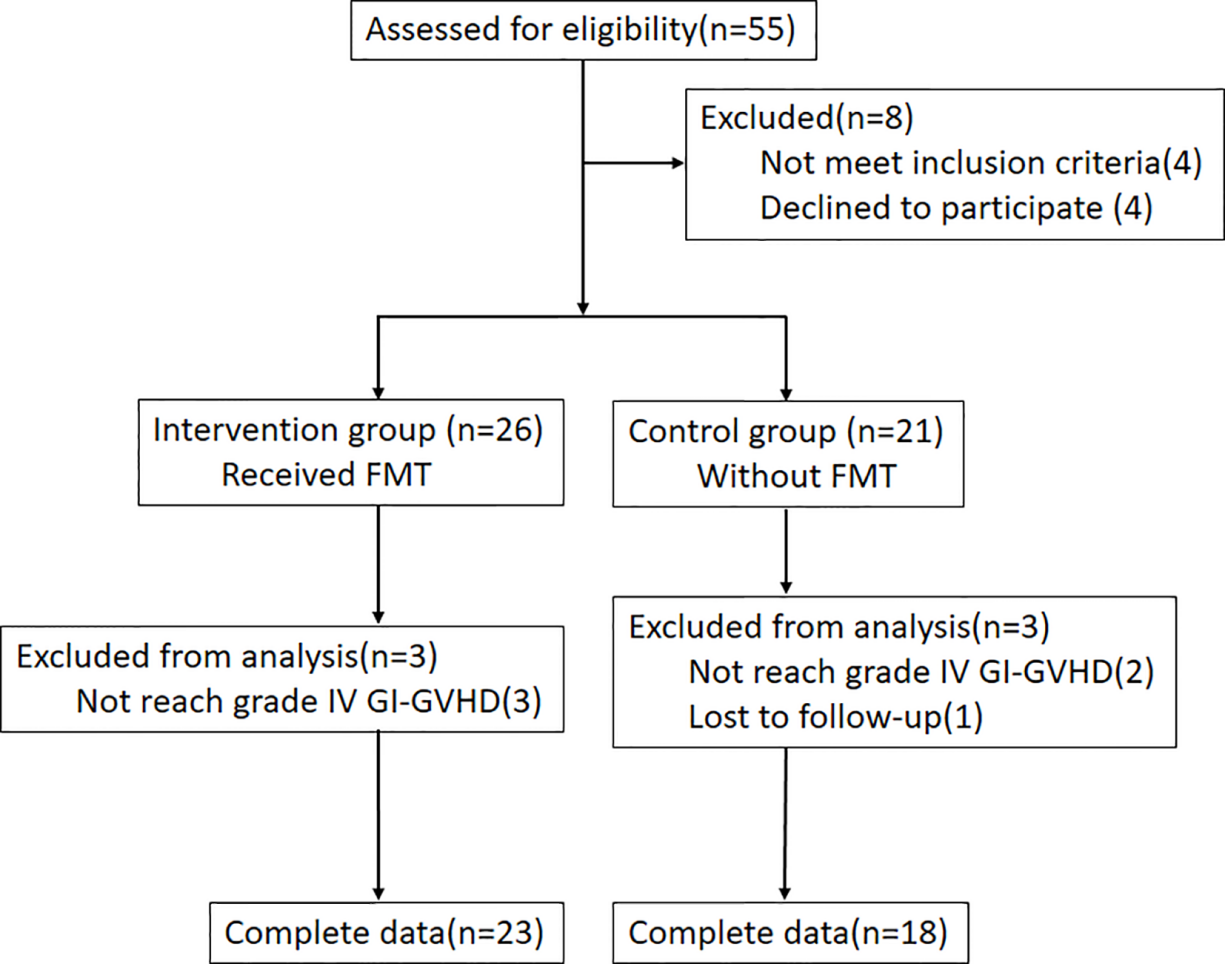
Experimental flow diagram.

A total of 55 patients with steroid-refractory GI-GvHD were enrolled in this study. *C. difficile* infection was not observed in any of the patients, and they did not respond to methylprednisolone (mPSL) at ≥2 mg/kg per day. Immunosuppressants as a second-line therapy were given to all the patients. Eight patients were excluded: Four patients were reluctant to participate in the study, while another four patients failed to meet the inclusion criteria (one for primary disease recurrence, two for combined thrombotic microangiopathy (TMA), and one for combined CMV before FMT). Of 26 patients in the FMT group, the data of three patients with < grade IV GI-GvHD were not selected for statistical analysis. Of the 21 patients in the control group, the data of three patients (one with missed follow-up and two with < grade IV GI-GvHD) were not used for statistical analysis (**Figure 1**).

The patients’ characteristics were shown in **Table 1** and **Supplementary Table 1**. Immunosuppressive drugs used were shown in **Supplementary Table 2**. The median age of the 23 patients in the FMT group was 30 years (range, 13–55 years). The male-to-female ratio was 16/7. The median stool volume was 660 mL/day (range, 360–2,080 mL/day). The median stool frequency was 6 times/day (range, 3–21 times/day). The median abdominal pain score was 3 (range, 1–4) (**Table 2** and **Supplementary Table 3**). For the 18 patients in the control group, the median age was 31.5 years (range, 13–59 years) (*vs.* FMT group, *p*>0.05). The male-to-female ratio was 7/11 (*vs.* FMT group, *p*>0.05). The median stool volume was 520 mL/day (range, 250–1,400 mL/day) (*vs.* FMT group, *p*<0.05). The median stool frequency was 5 times/day (range, 3–20 times/day) (*vs.* FMT group, *p*>0.05). The median abdominal pain score was 2 (range, 0–4) (*vs.* FMT group, *p*>0.05) (**Table 2** and **Supplementary Table 3**). No differences were observed in the occurrence of hematologic disease, stem cells donor gender match, or stem cell donor relationship between the two groups (**Table 1** and **Supplementary Table 1**). In the FMT group, 11, 9, 2, and 1 patients underwent 2, 1, 3, and 6 FMT sessions, respectively (**Supplementary Table 4**).

**Table 1:** Baseline characteristics of patients.

	FMT	Control	p
Median Age(min-max)	30(13-55)	31.5 (13-59)	>0.05
Gender			>0.05
male	16	7	
female	7	11	
Hematologic Disease			
AML	8	9	>0.05
ALL	3	2	>0.05
MDS	5	4	>0.05
AA	4	1	>0.05
CML	1	0	>0.05
Others	2	2	>0.05
Stem cells donor gender match	15	6	>0.05
Stem cells donor relationship			
Haplo-HSCT	20	14	>0.05
SIB-HSCT	1	3	>0.05
URD-HSCT	2	1	>0.05

**Table 2:** Clinical results of 14^th^ day and 21^st^ day.

		FMT (n=23)	Control (n=18)	p
0 day	Stool volume ml median(min-max)	660(360-2080)	520(250-1400)	<0.05^*^
	Stool frequencies	6(3-21)	5(3-20)	>0.05
	Abdominal pain score	3(1-4)	2(0-4)	>0.05
14^th^ day	Stool volume ml median(min-max)	200(0-1300)	500(0-1700)	<0.05^*^
	Stool frequencies	2(0-9)	5(0-12)	<0.05^*^
	Abdominal pain score	0(0-3)	2(0-4)	<0.05^*^
	CR	12(52.2%)	0(0%)	<0.05^*^
	Efficiency(CR+PR)	19(82.6%)	7(39%)	<0.05^*^
	Die	3(13.0%)	1(5.5%)	>0.05
21^st^ day	Stool volume ml median(min-max)	180(0-2365)	500(0-1400)	>0.05
	Stool frequencies	2(0-8)	4(0-15)	>0.05
	Abdominal pain score	0(0-3)	2(0-4)	>0.05
	CR	13(56.5%)	3(16%)	<0.05^*^
	Efficiency(CR+PR)	16(69.5%)	9(50%)	>0.05
	Die	3(13.0%)	2(11%)	>0.05
	GI-GvHD Relapse	2(8.6%)	2(11%)	>0.05

CR, clinical remission; PR, partial remission.

* means p<0.05.

Bold values for highlight p<0.05.

### Clinical Outcomes

Cox regression analysis showed that immunosuppressants did not affect the outcomes of the two groups (**Supplementary Table 2**).

**Figure 2:**
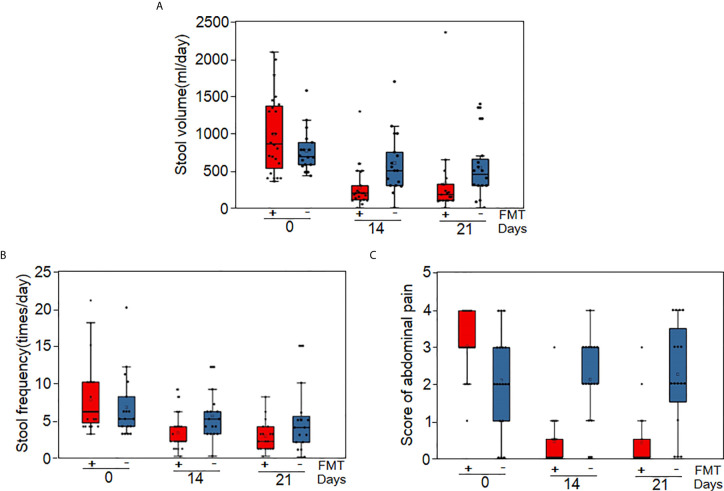
Clinical response to FMT. **(A)** Stool volumes of all patients at baseline, Day 14 and Day 21 after steroid-refractory GI-GvHD was diagnosed. **(B)** Stool frequency of all patients at baseline, Day 14 and Day 21 after steroid-refractory GI-GvHD was diagnosed. **(C)** Abdominal pain score of all patients at baseline, Day 14 and Day 21 after steroid-refractory GI-GvHD was diagnosed.

On day 14 (the day after the diagnosis of grade IV steroid-refractory GI-GvHD), 12 patients (52.2%) in the FMT group and none in the control group achieved clinical remission according to modified intention-to-treat analysis (*p*<0.05). Meanwhile, 19 patients (82.6%) in the FMT group and seven patients (39%) in the control group showed an effective response (clinical remission + partial remission) (RR=7.46; 95% CI, 1.78–31.4; *p*=0.006). Three patients (13.0%) in the FMT group and one patient (5.5%) in the control group died (RR=2.55; 95% CI, 0.24–26.84; p=0.436) (**Figure 2**, **Table 2**, and **Supplementary Table 3**). No relapse of GI-GvHD was recorded in any of the patients at this time point.

On day 21, clinical assessment showed that clinical remission was significantly more obvious in the FMT group than in the control group (13 [56.5%] of 23 patients *vs.* 3 [16%] of 18 patients; RR 9.36; 95% CI, 2.076–42.34; *p*=0.004), but the clinical response did not differ (16 [69.5%] of 23 patients *vs.* 9 [50%] of 18 patients; RR=2.83; 95% CI, 0.76–10.52; *p*=0.120). Three patients died in the FMT group, while two patients died in the control group (3 [13.0%] of 23 patients *vs.* 2 [11%] of 18 patients; RR=1.35; 95% CI, 0.20–9.02; *p*=0.57). Two patients in the FMT and control groups each showed GI-GvHD relapse, with no significant differences (2 [8.6%] of 23 patients *vs.* 2 [11%] of 18 patients; RR=0.857; 95% CI, 0.11–6.72; *p*=0.883) (**Table 2** and **Supplementary Table 3**). On day 28, the rate of clinical remission and effective response were higher in the FMT group than in the control group (**Supplementary Table 5**).

**Figure 3:**
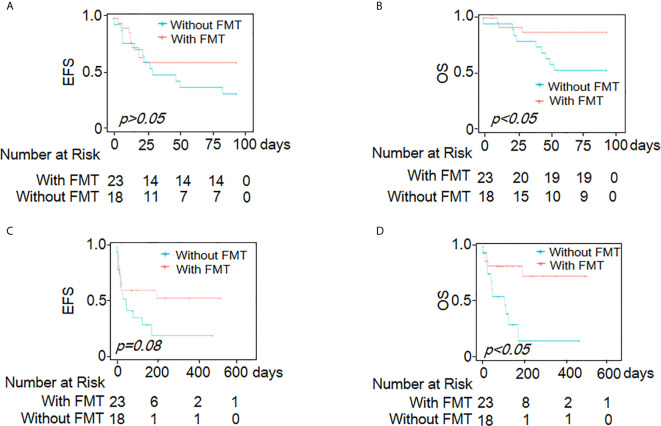
Kaplan-Meier curves demonstrating survival outcomes. EFS **(A)** and OS **(B)** of all patients within 90 days of follow-up time; EFS **(C)** and OS **(D)** at the end of research.

Within 90 days of follow-up, no significant difference was observed in EFS between the two groups (HR=1.8; 95% CI, 0.77–4.3; *p*=0.174) (**Figure 3A**). The FMT group showed better OS (HR=4.2; 95% CI, 1.1–16.0; *p*=0.031) (**Figure 3B** and **Supplementary Table 6**). At the end of the study, the median survival time was >539 days in the FMT group and 107 days in the control group (HR=3.51; 95% CI, 1.21–10.17; *p*=0.021). Both the EFS (HR=2.3; 95% CI, 0.99–5.4; *p*=0.08) and OS (HR= 4.4; 95% CI, 1.5–13.04; *p*=0.008) were higher in the FMT group than in the control group during the follow-up period (**Figures 3C, D** and **Supplementary Table 7**).

### Safety of FMT

Only one patient experienced thrombocytopenia after FMT, and one patient developed a cardiac event on the third day after FMT. It cannot be completely excluded that the occurrence of these two events was associated with FMT. No other severe adverse events were observed in the FMT group during 7 days of follow-up after FMT. Other common adverse events included an incomplete ileus in one patient, fever in one patient, vomiting and low fever in two patients, and grade-3 rash in two patients; these patients underwent symptomatic treatment.

Overall, the mortality rate was low in the FMT group (HR=3.97; 95% CI, 1.34–11.75; p=0.013). No significant differences were observed between the two groups in the occurrence of hemorrhagic cystitis (*p*>0.05), bacterial and fungal infection (*p*>0.05), CMV and EBV infection (*p*>0.05), septicemia (*p*>0.05), TMA (thrombotic microangiopathy) (*p*>0.05), cardiac events (*p*>0.05), thrombocytopenia (*p*>0.05), or epilepsy (*p*>0.05) (**Table 3** and **Supplementary Table 8**).

**Table 3:** Adverse events during overall follow-up time.

	Hemorrhagic cystitis	CMV & EBV	TMA	Infection rate (Bacteria & fungi)	Septicemia	Cardiac event	Thrombocytopenia & cerebral hemorrhage	Epilepsy	Die
FMT group (n=24)	1/23	8/23	5/23	5/23	2/23	2/23	1/23	1/23	5/23(21.7%)
Control group (n=18)	3/18	4/18	6/18	7/18	4/18	1/18	0/18	1/18	11/18(55.6%)
p	>0.05	>0.05	>0.05	>0.05	>0.05	>0.05	>0.05	>0.05	<0.05^*^

* means p<0.05.

Bold values for highlight p<0.05.

### Fecal Microbiota Analysis

**Figure 4:**
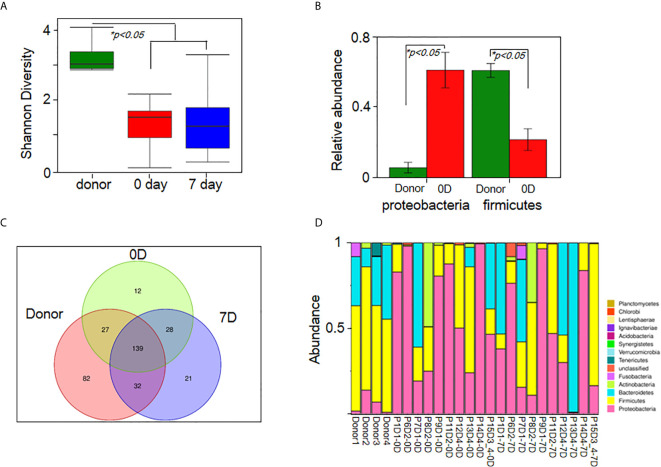
FMT improves gut microbiota diversity and composition in patients. **(A)** The diversity of fecal microbiota in all sample (Shannon’s diversity index)(n_donor_=4, n_patient_=10).**(B)** Relative abundance of *proteobacteria* and *firmicutes* between donor and recipient.*p < 0.05. **(C)** OTUs change in donor group, pre-FMT(0D) and post-FMT(7D) samples. **(D)** Analysis of fecal microbiota composition in all samples at the phylum level(n_donor_=4, n_patient_=10). Each row represents a study subject. Px means patient number, Dx means donor number, xD means day after FMT.

Given the severity and emergency of steroid-refractory grade IV GI-GvHD, only 10 patients provided fecal samples at baseline and at week 1 after FMT. Available fecal samples were subjected to microbiota analyses (N=10). Compared with that of the donors, the diversity of fecal microbiota in the fecal samples of the patients was lower before FMT (**Figure 4A**). The relative abundance of *Proteobacteria* increased, while that of *Firmicutes* decreased at the phylum level in the microbiota of the patients before FMT (**Figure 4B**). However, there was no significant difference before and after FMT because of the large variations between the patients. After week 1, the composition of microbiota was reconstructed in FMT patients and showed a trend back to normal (**Figures 4C, D**). Bacterial diversity improved at week 1 after FMT in half of the patients (5/10) (**Supplementary Table 4** and **Supplementary Figure S1A**). Similar to the results of our prior study, the ratio of *Firmicutes* to *Proteobacteria* was restored (7/10), the relative abundance of *Proteobacteria* decreased (9/10), and the relative abundance of *Firmicutes* increased (6/10) after FMT (**Supplementary Figure S1B**). The relative abundance of *Bacteroidetes* increased (7/10) in the fecal microbiota of patients with steroid-refractory GI-GvHD (**Supplementary Table 4** and **Supplementary Figure S1C**) after FMT.

## Discussion

Gut-GvHD related complications, especially steroid-refractory GI-GvHD, are one of causes of post-transplantation death (3–5), and the disruption of the gut microbiota has been linked to GvHD and transplantation-related mortality (24, 25).

FMT may restructure the gut microbiota of a patient and consequently reinforce the patient’s immune system (19). FMT has been proven very effective for the treatment of recurrent *C. difficile* infection (18) and other human diseases, such as inflammatory bowel disease (3, 16, 17, 23). Some case reports and our pilot study suggested that FMT may serve as a therapeutic option for steroid-refractory GI-GvHD (3, 14, 15, 20).

In this study, 55 patients with steroid-refractory GvHD of the gastrointestinal tract were enrolled. Twenty-three patients with grade IV steroid-refractory GI-GvHD received FMT (**Figure 1**). The microbial richness in terms of diversity and abundance showed an increasing trend after FMT in most of the patients compared with the patients’ gut microbiota before treatment, although there was no significant difference because of large variations. These results were similar to previous research (26). Moreover, the composition of the microbiota was restored. Beneficial bacteria, such as *Bacteroidetes* and *Firmicutes*, showed an increased trend after FMT in most of the patients (**Figure 4**).

In this study, diarrhea and abdominal pain were attenuated after FMT (**Figure 2**). The proportion of patients with clinical remission and effective response was higher at 2 or 3 weeks after FMT (**Table 2**). During a follow-up of 90 days, although EFS showed no significant difference between the two groups, the FMT group still showed better OS. Overall, the median survival in the FMT group was longer than that in the control group. Furthermore, both EFS and OS continued to increase during the follow-up in the FMT group (**Figure 3**).

In our study, only one case of thrombocytopenia, one case of cardiac event, and no cases of other severe adverse events were observed in the FMT group during the 7-day follow-up after FMT. Overall, FMT did not increase the probability of bacterial and fungal infections, CMV and EBV infections, or septicemia. The incidence rates of hemorrhagic cystitis, TMA, cardiac events, thrombocytopenia, and epilepsy were similar in the two groups (**Table 3**). Some studies reported that FMT transmitted drug-resistant *E. coli*, leading to patient death (27). No similar events were observed in our study, which may be attributed to our strict FMT donor criteria.

Although no data from any phase I/II clinical trial of FMT-treated GI-GvHD have been reported to date, our study was also limited in some aspects. First, it was conducted at a single institution, and thus, our findings may not be extrapolated directly to patients at other institutions (24). Second, given the severity and emergence of steroid-refractory grade IV GI-GvHD, the trial was not randomized and double-blind controlled. Moreover, the samples for gut microbiome analysis were procured from a limited number of patients; therefore, we could not study the gut microbiome dynamics in all patients, and, especially, we did not show the changes of the microbiome on days 14 and 21. Third, since immunosuppressants and antibiotics were administered, their effect on the gut microbiota could not be completely ruled out in this study. Fourth, not all of the patients showed similar responses to FMT, and more evidence of correcting GvHD-associated dysbiosis by FMT were needed in this study. We did not obtain enough data to compare the responding and non-responding patients due to the pathological complexity of steroid-refractory GI-GvHD.

In summary, although its effectiveness and safety need be verified in further studies, FMT may serve as a therapeutic option for grade IV steroid-refractory GI-GvHD.

## Data Availability Statement

The original contributions presented in the study are included in the article/**Supplementary Material**. Further inquiries can be directed to the corresponding authors.

## Ethics Statement

The studies involving human participants were reviewed and approved by the Institutional Review Board of the First Affiliated Hospital of Soochow University. The patients/participants provided their written informed consent to participate in this study.

## Author Contributions

XQ and DW contributed to the study concept and design. YZ, XL, and YJZ collected the clinical samples. YZ, XL, YJZ, and XQ performed the experiments. JG, YJ, and BZ performed bioinformatics analyses. XQ, XL, and YZ wrote the manuscript. DW supervised the study. All authors contributed to the article and approved the submitted version.

## Funding

This study was supported in part by grants from of National Key R&D Program of China (2017YFA0104502), Grants from of the National Science Foundation of China(8202010800), Translational Research Grant of NCRCH (2020ZKPC01), the Priority Academic Program Development of Jiangsu Higher Education Institutions (PAPD), Project of the State Key Laboratory of Radiation Medicine and Protection, Soochow University (GZN1202101) and “333 project” of Jiangsu (BRA2020398).

## Conflict of Interest

The authors declare that the research was conducted in the absence of any commercial or financial relationships that could be construed as a potential conflict of interest.

## Supplementary Material

Supplementary Figure 1 Analysis of fecal microbiota in donor and 10 patients. **(A)** The diversity of fecal microbiota(Shannon’s diversity index) change in pre-FMT(0D) and post-FMT(7D) samples. **(B)** Relative abundance of *proteobacteria* and *firmicutes* in donor group, pre-FMT(0D) and post-FMT(7D) samples. **p < 0.05.* **(C)** Relative abundance of *bacteroidetes* in pre-FMT(0D) and post-FMT(7D) samples.
