# Supplementary figures and images for: Safety and Efficacy of Fecal Microbiota Transplantation for Grade IV Steroid Refractory GI-GvHD Patients: Interim Results From FMT2017002 Trial
Source: Front Immunol. 2021 Jun 17;12:678476. doi: 10.3389/fimmu.2021.678476 (PMC8248496; doi:10.3389/fimmu.2021.678476)

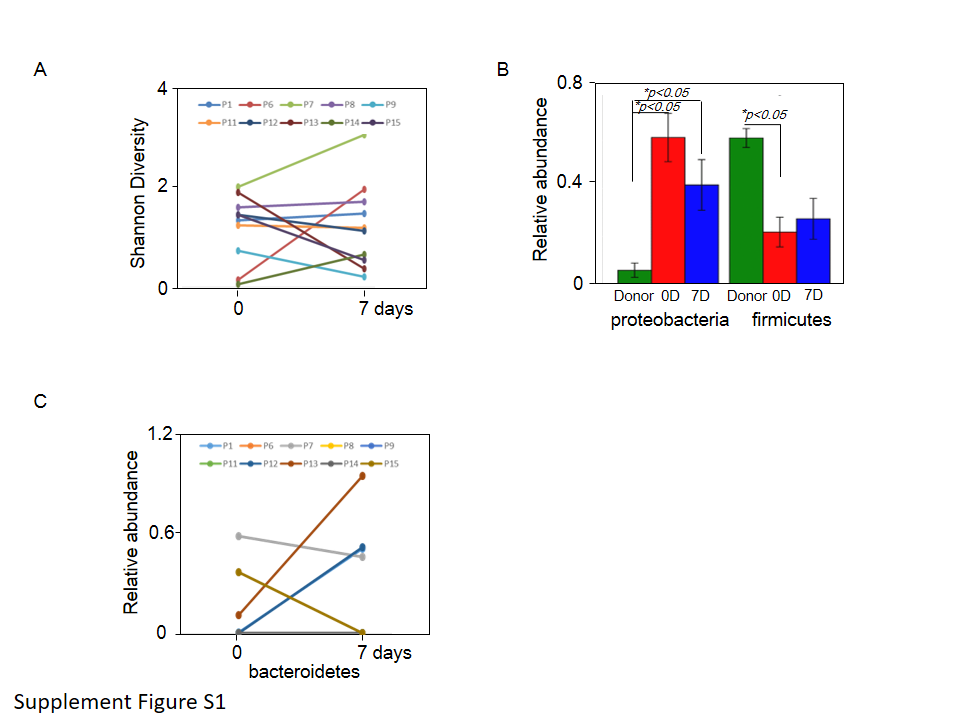

Supplement: Supplementary Figure 1 — Analysis of fecal microbiota in donor and 10 patients. (A) The diversity of fecal microbiota(Shannon’s diversity index) change in pre-FMT(0D) and post-FMT(7D) samples. (B) Relative abundance of proteobacteria and firmicutes in donor group, pre-FMT(0D) and post-FMT(7D) samples. *p < 0.05. (C) Relative abundance of bacteroidetes in pre-FMT(0D) and post-FMT(7D) samples. [file Image_1.tif]
