# Supplementary material for: Safety and Efficacy of Fecal Microbiota Transplantation for Grade IV Steroid Refractory GI-GvHD Patients: Interim Results From FMT2017002 Trial
Source: Front Immunol. 2021 Jun 17;12:678476. doi: 10.3389/fimmu.2021.678476 (PMC8248496; doi:10.3389/fimmu.2021.678476)
Supplement: Supplementary file 2 [file Table_1.docx]

Supplement table 1 **Baseline characteristics of all samples**

| number | gender | age | hematologic disease | Stem cells donor gender | Stem cells donor relationship | Stem cell source | Number of CD34+ x10^6^ content per kg recipient body weight | Diarrhea during pretreatment | Onset of GvHD (organ) | Onset of GvHD (days after alloHSCT) | Onset of GvHD (stage) | Onset of GI-GvHD (days after allo-HSCT) | skin-GvHD | liver-GvHD |
| --- | --- | --- | --- | --- | --- | --- | --- | --- | --- | --- | --- | --- | --- | --- |
| P1 | male | 13 | ALL | male | Haplo-HSCT | BM+PBSC | 1.98 | 0 | GI,skin | 302 | IV | 302 | 1 | 0 |
| P10 | male | 47 | MDS | male | Haplo-HSCT | PBSC | 2.5 | 0 | skin | 28 | I | 31 | 1 | 0 |
| P11 | female | 30 | AML | male | Haplo-HSCT | Cord Blood+ BM+PBSC | 3.94 | 0 | GI | 173 | IV | 173 | 0 | 0 |
| P12 | male | 33 | AML | male | Haplo-HSCT | Cord Blood+ BM+PBSC | 2.989 | 1 | skin | 15 | I | 27 | 1 | 0 |
| P13 | male | 21 | AML | male | Haplo-HSCT | Cord Blood+ BM+PBSC | 3.04 | 0 | skin | 11 | I | 504 | 1 | 0 |
| P14 | male | 43 | AML | male | URD-HSCT | PBSC | 12 | 0 | GI | 22 | IV | 22 | 0 | 0 |
| P15 | male | 26 | AML | female | Haplo-HSCT | PBSC | 2 | 0 | skin | 139 | I | 142 | 1 | 0 |
| P16 | male | 38 | AML | male | Haplo-HSCT | PBSC | 3.29 | 1 | GI | 52 | IV | 52 | 0 | 0 |
| P17 | female | 21 | ALL | male | Haplo-HSCT | BM+PBSC | 3.5 | 1 | skin | 41 | III | 43 | 1 | 1 |
| P18 | female | 28 | ALL | female | Haplo-HSCT | BM | 4.97 | 1 | skin | 30 | I | 33 | 1 | 0 |
| P19 | male | 16 | AA | male | Haplo-HSCT | BM+PBSC | 2.925 | 0 | GI | 19 | IV | 19 | 0 | 0 |
| P2 | male | 23 | AA | male | Haplo-HSCT | Cord Blood+ BM+PBSC | 3.92 | 1 | GI | 181 | IV | 181 | 0 | 0 |
| P20 | male | 20 | AML | male | URD-HSCT | PBSC | 4.58 | 1 | skin | 357 | I | 369 | 1 | 1 |
| P21 | male | 42 | DLBCL | male | Haplo-HSCT | PBSC | 4.52 | 0 | GI,live | 78 | IV | 78 | 0 | 1 |
| P22 | male | 48 | CML | female | Haplo-HSCT | BM+PBSC | 1.99 | 1 | GI | 79 | IV | 79 | 0 | 0 |
| P23 | male | 17 | AA | male | Haplo-HSCT | Cord Blood+ BM+PBSC | 1.628 | 1 | skin | 15 | I | 21 | 1 | 1 |
| P3 | male | 55 | MDS | male | Haplo-HSCT | BM+PBSC | 4.5 | 1 | GI | 18 | IV | 18 | 0 | 0 |
| P4 | female | 23 | AML | male | Haplo-HSCT | Cord Blood+ BM+PBSC | 4.43 | 1 | GI | 23 | IV | 23 | 0 | 0 |
| P5 | male | 47 | MDS | male | Haplo-HSCT | Cord Blood+ BM+PBSC | 5.84 | 0 | GI,skin | 34 | IV | 34 | 1 | 0 |
| P6 | female | 42 | HAL | male | Haplo-HSCT | BM+PBSC | 2.38 | 1 | skin | 25 | I | 27 | 1 | 0 |
| P7 | female | 14 | AA | male | Haplo-HSCT | Cord Blood+ BM+PBSC | 2.5 | 0 | GI | 41 | IV | 41 | 0 | 0 |
| P8 | female | 52 | MDS | male | SIB-HSCT | PBSC | 5.3 | 0 | GI | 90 | IV | 90 | 0 | 0 |
| P9 | male | 17 | MDS | male | Haplo-HSCT | BM+PBSC | 2.83 | 1 | GI | 31 | IV | 31 | 0 | 0 |
| PC1 | female | 31 | AML | male | Haplo-HSCT | BM+PBSC | 1.82 | 1 | skin | 21 | I | 30 | 1 | 0 |
| PC2 | female | 14 | MDS | male | Haplo-HSCT | PBSC | 5.3 | 0 | skin,liver,GI | 21 | IV | 21 | 1 | 1 |
| PC3 | female | 49 | IMF | male | Haplo-HSCT | Cord Blood+ BM+PBSC | 5.35 | 1 | GI | 151 | IV | 151 | 0 | 0 |
| PC4 | male | 26 | ALL | male | Haplo-HSCT | Cord Blood+ BM+PBSC | 4.06 | 0 | skin | 19 | I | 22 | 1 | 0 |
| PC5 | female | 49 | AML | male | Haplo-HSCT | BM+PBSC | 3.3 | 0 | GI,liver | 35 | IV | 35 | 0 | 1 |
| PC6 | female | 39 | AA | male | Haplo-HSCT | BM+PBSC | 3.5 | 1 | skin | 18 | I | 21 | 1 | 0 |
| PC7 | female | 23 | AML | male | Haplo-HSCT | BM+PBSC | 2.89 | 0 | skin | 37 | III | 45 | 1 | 0 |
| PC8 | male | 32 | AML | male | Haplo-HSCT | BM+PBSC | 6.9 | 0 | GI | 45 | IV | 45 | 0 | 0 |
| PC9 | female | 51 | AML | female | Haplo-HSCT | Cord Blood+ BM+PBSC | 3.13 | 1 | skin | 25 | I | 27 | 1 | 0 |
| PC10 | male | 29 | AML | male | SIB-HSCT | PBSC | 2.8 | 0 | GI | 144 | IV | 144 | 0 | 0 |
| PC11 | male | 45 | AML | female | Haplo-HSCT | BM+PBSC | 3.751 | 0 | skin | 25 | I | 110 | 1 | 0 |
| PC12 | male | 16 | MDS | female | Haplo-HSCT | Cord Blood+ BM+PBSC | 3.56 | 1 | skin | 13 | II | 28 | 1 | 0 |
| PC13 | female | 41 | AML | male | SIB-HSCT | PBSC | 3.12 | 0 | skin | 31 | III | 38 | 1 | 0 |
| PC14 | female | 48 | MDS | male | SIB-HSCT | PBSC | 1.95 | 0 | skin | 22 | I | 87 | 1 | 1 |
| PC15 | male | 30 | TLBL | male | URD-HSCT | PBSC | 4.15 | 1 | GI | 74 | III | 74 | 0 | 0 |
| PC16 | female | 59 | MDS | male | Haplo-HSCT | Cord Blood+ BM+PBSC | 2.3 | 1 | GI | 33 | IV | 33 | 0 | 0 |
| PC17 | female | 19 | AML | male | Haplo-HSCT | Cord Blood+ BM+PBSC | 2.535 | 1 | liver、GI | 102 | V | 102 | 0 | 1 |
| PC18 | male | 13 | ALL | male | Haplo-HSCT | Cord Blood+ BM+PBSC | 3.44 | 0 | skin | 16 | II | 18 | 1 | 0 |
