# Supplementary material for: Safety and Efficacy of Fecal Microbiota Transplantation for Grade IV Steroid Refractory GI-GvHD Patients: Interim Results From FMT2017002 Trial
Source: Front Immunol. 2021 Jun 17;12:678476. doi: 10.3389/fimmu.2021.678476 (PMC8248496; doi:10.3389/fimmu.2021.678476)
Supplement: Supplementary file 3 [file Table_2.docx]

Supplement table 2 **Immunosuppressive drugs use for all samples**

| number | methylprednisolone | budesonide | etanercept | basiliximab | tacrolimus | cyclophosphamide | cyclosporin | Ruxolitinib | MTX | mycophenolate |
| --- | --- | --- | --- | --- | --- | --- | --- | --- | --- | --- |
| P1 | 1 | 0 | 0 | 0 | 1 | 0 | 1 | 1 | 1 | 0 |
| P10 | 1 | 0 | 1 | 1 | 1 | 1 | 1 | 0 | 1 | 1 |
| P11 | 1 | 0 | 0 | 0 | 1 | 0 | 1 | 0 | 0 | 0 |
| P12 | 1 | 0 | 1 | 1 | 1 | 1 | 1 | 1 | 1 | 1 |
| P13 | 1 | 0 | 1 | 1 | 1 | 0 | 1 | 0 | 1 | 0 |
| P14 | 1 | 1 | 1 | 1 | 1 | 1 | 1 | 0 | 0 | 0 |
| P15 | 1 | 0 | 1 | 1 | 1 | 1 | 1 | 1 | 0 | 0 |
| P16 | 1 | 0 | 0 | 1 | 1 | 0 | 1 | 1 | 0 | 0 |
| P17 | 1 | 0 | 1 | 1 | 1 | 0 | 1 | 1 | 0 | 0 |
| P18 | 1 | 0 | 0 | 1 | 1 | 0 | 1 | 0 | 0 | 0 |
| P19 | 1 | 1 | 0 | 1 | 1 | 1 | 1 | 1 | 1 | 1 |
| P2 | 1 | 0 | 0 | 0 | 1 | 0 | 1 | 0 | 0 | 1 |
| P20 | 1 | 0 | 0 | 0 | 1 | 1 | 1 | 0 | 0 | 0 |
| P21 | 1 | 0 | 1 | 0 | 0 | 1 | 1 | 0 | 1 | 1 |
| P22 | 1 | 0 | 0 | 0 | 1 | 1 | 1 | 1 | 0 | 0 |
| P23 | 1 | 0 | 1 | 1 | 1 | 0 | 1 | 0 | 0 | 1 |
| P3 | 1 | 1 | 0 | 1 | 1 | 1 | 1 | 0 | 1 | 1 |
| P4 | 1 | 0 | 0 | 1 | 1 | 1 | 1 | 1 | 0 | 0 |
| P5 | 1 | 1 | 0 | 1 | 1 | 1 | 1 | 0 | 0 | 0 |
| P6 | 1 | 0 | 0 | 1 | 1 | 1 | 1 | 0 | 0 | 0 |
| P7 | 1 | 1 | 0 | 0 | 1 | 1 | 1 | 0 | 0 | 0 |
| P8 | 1 | 0 | 0 | 0 | 0 | 0 | 1 | 0 | 0 | 1 |
| P9 | 1 | 1 | 1 | 1 | 1 | 1 | 1 | 0 | 1 | 0 |
| PC1 | 1 | 1 | 1 | 1 | 1 | 1 | 1 | 1 | 1 | 1 |
| PC2 | 1 | 1 | 0 | 1 | 1 | 1 | 1 | 1 | 0 | 1 |
| PC3 | 1 | 0 | 1 | 1 | 1 | 1 | 1 | 0 | 0 | 1 |
| PC4 | 1 | 0 | 1 | 1 | 1 | 0 | 1 | 0 | 1 | 1 |
| PC5 | 1 | 0 | 0 | 1 | 1 | 0 | 1 | 1 | 1 | 1 |
| PC6 | 1 | 1 | 1 | 1 | 1 | 1 | 1 | 0 | 0 | 1 |
| PC7 | 1 | 0 | 1 | 1 | 1 | 0 | 1 | 1 | 1 | 1 |
| PC8 | 1 | 1 | 0 | 1 | 1 | 0 | 1 | 0 | 1 | 1 |
| PC9 | 1 | 0 | 0 | 1 | 1 | 0 | 1 | 0 | 1 | 1 |
| PC10 | 1 | 0 | 0 | 1 | 1 | 0 | 1 | 1 | 1 | 1 |
| PC11 | 1 | 1 | 0 | 1 | 1 | 0 | 1 | 1 | 0 | 1 |
| PC12 | 1 | 1 | 0 | 1 | 1 | 0 | 1 | 0 | 0 | 1 |
| PC13 | 1 | 0 | 0 | 0 | 1 | 0 | 1 | 0 | 0 | 0 |
| PC14 | 1 | 0 | 1 | 1 | 1 | 1 | 1 | 0 | 1 | 0 |
| PC15 | 1 | 0 | 0 | 0 | 1 | 1 | 1 | 0 | 1 | 1 |
| PC16 | 1 | 1 | 1 | 1 | 1 | 1 | 1 | 0 | 1 | 1 |
| PC17 | 1 | 0 | 1 | 1 | 1 | 0 | 1 | 0 | 1 | 0 |
| PC18 | 1 | 0 | 1 | 1 | 1 | 0 | 1 | 0 | 1 | 0 |
