# Supplementary material for: Safety and Efficacy of Fecal Microbiota Transplantation for Grade IV Steroid Refractory GI-GvHD Patients: Interim Results From FMT2017002 Trial
Source: Front Immunol. 2021 Jun 17;12:678476. doi: 10.3389/fimmu.2021.678476 (PMC8248496; doi:10.3389/fimmu.2021.678476)
Supplement: Supplementary file 4 [file Table_3.docx]

Supplement table 3 **Clinic characteristics of all samples within 0D, 14D, 21D**

| number | stool volume (ml/day)0D | stool volume (ml/day)14D | stool volume (ml/day)21D | stool frequency (times/day)0D | stool frequency (times/day)14D | stool frequency (times/day)21D | Abdominal pain score 0D | Abdominal pain score 14D | Abdominal pain score 21D | GI-GvHD clinical outcome in 14D | survival in 14D | GI-GvHD clinical outcome in 21D | survival in 21D. | relapse in 21D. |
| --- | --- | --- | --- | --- | --- | --- | --- | --- | --- | --- | --- | --- | --- | --- |
| P1 | 400 | 50 | 100 | 5 | 1 | 2 | 3 | 0 | 0 | 2 | 1 | 2 | 1 | 0 |
| P10 | 1350 | die | die | 6 | die | die | 4 | die | die | 0 | 0 | 0 | 0 | 0 |
| P11 | 400 | 300 | 100 | 4 | 4 | 1 | 3 | 0 | 0 | 1 | 1 | 2 | 1 | 0 |
| P12 | 600 | 110 | 180 | 6 | 2 | 3 | 4 | 1 | 1 | 1 | 1 | 1 | 1 | 0 |
| P13 | 700 | 200 | 100 | 6 | 3 | 2 | 3 | 0.5 | 0 | 1 | 1 | 1 | 1 | 0 |
| P14 | 1300 | 1300 | 2365 | 15 | 9 | 8 | 2 | 0 | 0 | 0 | 1 | 0 | 1 | 0 |
| P15 | 1450 | 200 | 210 | 10 | 2 | 3 | 3 | 0 | 0 | 2 | 1 | 2 | 1 | 0 |
| P16 | 690 | 0 | 150 | 6 | 0 | 1 | 3 | 0.5 | 0 | 2 | 1 | 2 | 1 | 0 |
| P17 | 1790 | 130 | 315 | 18 | 6 | 3 | 3 | 3 | 3 | 2 | 1 | 2 | 1 | 0 |
| P18 | 1250 | 230 | 200 | 8 | 2 | 1 | 4 | 1 | 0 | 2 | 1 | 2 | 1 | 0 |
| P19 | 400 | 180 | 320 | 5 | 2 | 4 | 2 | 0 | 0 | 1 | 1 | 0 | 1 | 1 |
| P2 | 1000 | 100 | 0 | 6 | 1 | 0 | 4 | 0 | 0 | 2 | 1 | 0 | 1 | 0 |
| P20 | 2080 | die | die | 11 | die | die | 4 | die | die | 0 | 0 | 0 | 0 | 0 |
| P21 | 400 | die | die | 4 | die | die | 4 | die | die | 0 | 0 | 0 | 0 | 0 |
| P22 | 880 | 100 | 100 | 4 | 2 | 1 | 2 | 0 | 0 | 2 | 1 | 2 | 1 | 0 |
| P23 | 800 | 100 | 90 | 4 | 1 | 1 | 3 | 0 | 0 | 2 | 1 | 2 | 1 | 0 |
| P3 | 1300 | 595 | 400 | 10 | 6 | 5 | 1 | 0 | 0 | 2 | 1 | 2 | 1 | 0 |
| P4 | 2000 | 500 | 230 | 10 | 4 | 2 | 3 | 1 | 1 | 2 | 1 | 2 | 1 | 0 |
| P5 | 465 | 210 | 100 | 4 | 8 | 3 | 3 | 1 | 0.5 | 1 | 1 | 1 | 1 | 0 |
| P6 | 1500 | 500 | 500 | 21 | 4 | 4 | 3 | 0 | 0 | 2 | 1 | 2 | 1 | 0 |
| P7 | 850 | 150 | 0 | 6 | 2 | 0 | 4 | 0.5 | 0 | 2 | 1 | 2 | 1 | 0 |
| P8 | 360 | 200 | 200 | 3 | 2 | 1 | 3 | 0 | 0 | 1 | 1 | 2 | 1 | 0 |
| P9 | 660 | 485 | 650 | 6 | 4 | 6 | 2 | 0.5 | 2 | 1 | 1 | 0 | 1 | 1 |
| PC1 | 750 | 1000 | 500 | 4 | 9 | 5 | 0 | 3 | 2 | 0 | 1 | 1 | 1 | 0 |
| PC2 | 900 | 1100 | 1200 | 10 | 12 | 15 | 3 | 2 | 2 | 0 | 1 | 0 | 1 | 0 |
| PC3 | 1000 | 1000 | 1400 | 12 | 12 | 15 | 4 | 3 | 3 | 0 | 1 | 0 | 1 | 0 |
| PC4 | 600 | 300 | 550 | 5 | 3 | 5 | 2 | 0 | 0 | 1 | 1 | 1 | 1 | 0 |
| PC5 | 500 | 500 | die | 4 | 4 | die | 2 | 2 | die | 0 | 1 | 0 | 0 | 0 |
| PC6 | 400 | 750 | 700 | 3 | 7 | 6 | 2 | 0 | 1 | 0 | 1 | 0 | 1 | 0 |
| PC7 | 400 | 550 | 400 | 4 | 6 | 4 | 2 | 4 | 4 | 0 | 1 | 0 | 1 | 0 |
| PC8 | 300 | 600 | 100 | 3 | 6 | 1 | 0 | 2 | 2 | 0 | 1 | 2 | 1 | 0 |
| PC9 | 600 | 700 | 300 | 5 | 5 | 2 | 3 | 3 | 2 | 0 | 1 | 1 | 1 | 0 |
| PC10 | 300 | 200 | 80 | 6 | 4 | 1 | 3 | 3 | 2 | 1 | 1 | 2 | 1 | 0 |
| PC11 | 600 | 350 | 300 | 5 | 3 | 3 | 4 | 2 | 4 | 1 | 1 | 1 | 1 | 0 |
| PC12 | 250 | 300 | 300 | 3 | 3 | 3 | 4 | 2 | 4 | 0 | 1 | 0 | 1 | 0 |
| PC13 | 700 | 500 | 500 | 6 | 4 | 4 | 1 | 1 | 0 | 1 | 1 | 1 | 1 | 0 |
| PC14 | 460 | die | die | 11 | die | die | 3 | die | die | 0 | 0 | 0 | 0 | 0 |
| PC15 | 500 | 290 | 1350 | 3 | 3 | 10 | 0 | 2 | 3 | 1 | 1 | 0 | 1 | 1 |
| PC16 | 520 | 390 | 610 | 6 | 5 | 5 | 2 | 3 | 4 | 1 | 1 | 0 | 1 | 1 |
| PC17 | 380 | 0 | 0 | 8 | 0 | 0 | 2 | 3 | 3 | 1 | 1 | 1 | 1 | 0 |
| PC18 | 1400 | 1700 | 310 | 20 | 6 | 2 | 1 | 1 | 0 | 0 | 1 | 2 | 1 | 0 |

GI-GvHD clinical outcome：clinical remission=2; partial remission=1;no response=0；survival. survival=1; death=0；relapse：relapse=1; without relapse=0
