# Supplementary material for: Safety and Efficacy of Fecal Microbiota Transplantation for Grade IV Steroid Refractory GI-GvHD Patients: Interim Results From FMT2017002 Trial
Source: Front Immunol. 2021 Jun 17;12:678476. doi: 10.3389/fimmu.2021.678476 (PMC8248496; doi:10.3389/fimmu.2021.678476)
Supplement: Supplementary file 5 [file Table_4.docx]

Supplement table 4 **Donor or times and Change of fecal microbiota for all samples**

| number | donor | times | Proteobacteria 0D | Proteobacteria 7D | Firmicutes 0D | Firmicutes 7D | Firmicutes/Proteobacteria 0D | Firmicutes/Proteobacteria 7D | Bacteroidetes 0D | Bacteroidetes 7D | Shannon's diversity index 0D | Shannon's diversity index 7D |
| --- | --- | --- | --- | --- | --- | --- | --- | --- | --- | --- | --- | --- |
| P1 | D1 | 2 | 0.828189048 | 0.379487328 | 0.16228147 | 0.087281796 | 0.195947375 | 0.229999236 | 0.006481449 | 0.531375051 | 1.438053 | 1.586007 |
| P10 | D1 | 2 | / | / | / | / | / | / | / | / | / | / |
| P11 | D2 | 2 | 0.875521822 | 0.469327498 | 0.118817204 | 0.523377796 | 0.135710157 | 1.115165421 | 0.000189753 | 0.00020842 | 1.332666 | 1.28216 |
| P12 | D4 | 3 | 0.501204981 | 0.299595526 | 0.485406346 | 0.160494217 | 0.968478696 | 0.535702985 | 0.000267773 | 0.539562662 | 1.561814 | 1.209932 |
| P13 | D4 | 2 | 0.239717491 | 0.006680221 | 0.618993214 | 0.003485333 | 2.582177932 | 0.52173913 | 0.113453815 | 0.988672669 | 2.038732 | 0.395925 |
| P14 | D4 | 6 | 0.993425005 | 0.837125263 | 0.000367132 | 0.161952874 | 0.000369562 | 0.193463131 | 0.000166878 | 0.000368745 | 0.058953 | 0.710429 |
| P15 | D3+D4 | 3 | 0.464883902 | 0.164519187 | 0.148368074 | 0.829582558 | 0.31915081 | 5.042466923 | 0.383717597 | 0.000561739 | 1.561583 | 0.58475 |
| P16 | D1 | 1 | / | / | / | / | / | / | / | / | / | / |
| P17 | D3 | 1 | / | / | / | / | / | / | / | / | / | / |
| P18 | D1 | 1 | / | / | / | / | / | / | / | / | / | / |
| P19 | D3 | 1 | / | / | / | / | / | / | / | / | / | / |
| P2 | D2 | 2 | / | / | / | / | / | / | / | / | / | / |
| P20 | D1 | 1 | / | / | / | / | / | / | / | / | / | / |
| P21 | D3 | 1 | / | / | / | / | / | / | / | / | / | / |
| P22 | D1 | 2 | / | / | / | / | / | / | / | / | / | / |
| P23 | D3 | 1 | / | / | / | / | / | / | / | / | / | / |
| P3 | D1 | 1 | / | / | / | / | / | / | / | / | / | / |
| P4 | D1 | 2 | / | / | / | / | / | / | / | / | / | / |
| P5 | D2 | 1 | / | / | / | / | / | / | / | / | / | / |
| P6 | D4 | 2 | 0.979693742 | 0.762814254 | 0.006748578 | 0.128051013 | 0.006888456 | 0.167866571 | 0.000605254 | 0.002837442 | 0.158269 | 2.110262 |
| P7 | D1 | 2 | 0.191582585 | 0.155526551 | 0.1982474 | 0.264752353 | 1.034788211 | 1.70229682 | 0.608472955 | 0.48073092 | 2.161289 | 3.285355 |
| P8 | D2 | 2 | 0.249294118 | 0.108912479 | 0.258039216 | 0.54084218 | 1.03507944 | 4.965842167 | 0.001647059 | 0.001058337 | 1.716173 | 1.838375 |
| P9 | D1 | 2 | 0.804748097 | 0.964225352 | 0.179630301 | 0.032887324 | 0.223213079 | 0.034107508 | 0.00079739 | 0.000211268 | 0.781206 | 0.220453 |
