# Supplementary material for: Safety and Efficacy of Fecal Microbiota Transplantation for Grade IV Steroid Refractory GI-GvHD Patients: Interim Results From FMT2017002 Trial
Source: Front Immunol. 2021 Jun 17;12:678476. doi: 10.3389/fimmu.2021.678476 (PMC8248496; doi:10.3389/fimmu.2021.678476)
Supplement: Supplementary file 6 [file Table_5.docx]

Supplement table 5 Clinical results of 28^th^ day

|  |  | FMT  (n=23) | Control  (n=18) | p |
| --- | --- | --- | --- | --- |
| 28^th^ day | CR | 18(78.3%) | 6(33.3%) | **<0.05*** |
|  | Efficiency(CR+PR) | 20(87.0%) | 11(61.1%) | **<0.05*** |
|  | Die | 3(13.0%) | 3(16.6%) | >0.05 |

CR(clinical remission); PR(partial remission)
