# Supplementary material for: Safety and Efficacy of Fecal Microbiota Transplantation for Grade IV Steroid Refractory GI-GvHD Patients: Interim Results From FMT2017002 Trial
Source: Front Immunol. 2021 Jun 17;12:678476. doi: 10.3389/fimmu.2021.678476 (PMC8248496; doi:10.3389/fimmu.2021.678476)
Supplement: Supplementary file 7 [file Table_6.docx]

Supplement table 6 **Clinical results within 90 days follow-up time (after steroid-refractory GI-GvHD was diagnosed)**

| number | Events within 90 days | Event time within 90 days | survival time during 90 days follow-up time | survival in 90 days  survival=1; death=0 |
| --- | --- | --- | --- | --- |
| P1 | CMV | 19 | 90 | 1 |
| P10 | die | 12 | 12 | 0 |
| P11 | no | 90 | 90 | 1 |
| P12 | no | 90 | 90 | 1 |
| *P13 | no | 90 | 90 | 1 |
| *P14 | CMV | 19 | 90 | 1 |
| *P15 | no | 90 | 90 | 1 |
| P16 | no | 90 | 90 | 1 |
| P17 | GI worsen | 4 | 90 | 1 |
| P18 | no | 90 | 90 | 1 |
| P19 | CMV | 22 | 90 | 1 |
| P2 | no | 90 | 90 | 1 |
| P20 | die | 11 | 11 | 0 |
| P21 | die | 7 | 7 | 0 |
| *P22 | no | 90 | 90 | 1 |
| P23 | no | 90 | 90 | 1 |
| P3 | no | 90 | 90 | 1 |
| P4 | CMV | 13 | 29 | 0 |
| P5 | no | 90 | 90 | 1 |
| P6 | no | 90 | 90 | 1 |
| P7 | CMV | 13 | 90 | 1 |
| P8 | no | 90 | 90 | 1 |
| P9 | CMV | 14 | 90 | 1 |
| PC1 | CMV | 6 | 52 | 0# |
| PC2 | TMA | 7 | 39 | 0# |
| *PC3 | no | 90 | 90 | 1 |
| PC4 | die | 49 | 49 | 0 |
| PC5 | die | 22 | 22 | 0 |
| PC6 | TMA | 29 | 43 | 0 |
| PC7 | TMA | 16 | 25 | 0 |
| PC8 | no | 90 | 90 | 1 |
| PC9 | CMV | 7 | 90 | 1 |
| *PC10 | no | 90 | 90 | 1 |
| PC11 | TMA | 80 | 90 | 1 |
| PC12 | CMV | 27 | 90 | 1 |
| PC13 | die | 46 | 46 | 0 |
| PC14 | die | 1 | 1 | 0 |
| PC15 | no | 90 | 90 | 1 |
| PC16 | die | 23 | 23 | 0 |
| PC17 | no | 90 | 90 | 1 |
| PC18 | no | 90 | 90 | 1 |

# patients death in 90 days after steroid-refractory GI-GvHD was diagnosed but after November 2018(end of follow up time)

* 90 days after steroid-refractory GI-GvHD was diagnosed but after November 2018(end of follow up time)
