# Supplementary material for: Safety and Efficacy of Fecal Microbiota Transplantation for Grade IV Steroid Refractory GI-GvHD Patients: Interim Results From FMT2017002 Trial
Source: Front Immunol. 2021 Jun 17;12:678476. doi: 10.3389/fimmu.2021.678476 (PMC8248496; doi:10.3389/fimmu.2021.678476)
Supplement: Supplementary file 8 [file Table_7.docx]

Supplement table 7 **Clinical results at the end of research (after steroid-refractory GI-GvHD was diagnosed)**

| number | Events in overall time | Event time | survival time at the end of research | survival at the end of research  survival=1; death=0 |
| --- | --- | --- | --- | --- |
| P1 | CMV | 19 | 145 | 1 |
| P10 | die | 12 | 12 | 0 |
| P11 | no | 239 | 239 | 1 |
| P12 | no | 102 | 102 | 1 |
| P13 | no | 76 | 76 | 1 |
| P14 | CMV | 19 | 75 | 1 |
| P15 | no | 71 | 71 | 1 |
| P16 | no | 180 | 180 | 1 |
| P17 | GI worsen | 4 | 120 | 1 |
| P18 | no | 490 | 490 | 1 |
| P19 | CMV | 22 | 90 | 1 |
| P2 | no | 232 | 232 | 1 |
| P20 | die | 11 | 11 | 0 |
| P21 | die | 7 | 7 | 0 |
| P22 | no | 82 | 82 | 1 |
| P23 | no | 93 | 93 | 1 |
| P3 | die | 192 | 192 | 0 |
| P4 | CMV | 13 | 29 | 0 |
| P5 | no | 356 | 356 | 1 |
| P6 | no | 504 | 504 | 1 |
| P7 | CMV | 13 | 356 | 1 |
| P8 | no | 348 | 348 | 1 |
| P9 | CMV | 14 | 293 | 1 |
| PC1 | CMV | 6 | 31 | 1 |
| PC2 | TMA | 7 | 18 | 1 |
| PC3 | no | 7 | 7 | 1 |
| PC4 | die | 49 | 49 | 0 |
| PC5 | die | 22 | 22 | 0 |
| PC6 | TMA | 29 | 43 | 0 |
| PC7 | TMA | 16 | 25 | 0 |
| PC8 | no | 148 | 148 | 1 |
| PC9 | CMV | 7 | 113 | 0 |
| PC10 | no | 77 | 77 | 1 |
| PC11 | TMA | 80 | 107 | 0 |
| PC12 | CMV | 27 | 120 | 1 |
| PC13 | die | 46 | 46 | 0 |
| PC14 | die | 1 | 1 | 0 |
| PC15 | die | 171 | 171 | 0 |
| PC16 | die | 23 | 23 | 0 |
| PC17 | die | 125 | 125 | 0 |
| PC18 | no | 464 | 464 | 1 |
