# Supplementary material for: Safety and Efficacy of Fecal Microbiota Transplantation for Grade IV Steroid Refractory GI-GvHD Patients: Interim Results From FMT2017002 Trial
Source: Front Immunol. 2021 Jun 17;12:678476. doi: 10.3389/fimmu.2021.678476 (PMC8248496; doi:10.3389/fimmu.2021.678476)
Supplement: Supplementary file 10 [file DataSheet_1.docx]

**Supplemental Subjects and Methods**

**Study design and participants**

We undertook an open-label, non-randomized, phase I/II clinical study at the First Affiliated Hospital of Soochow University. Protocols and other trial related procedures were approved by the Institutional Review Board of the Hospital. All the patients signed written informed consent. FMT group and control group (without FMT) were set according the patients decision after introducing the possible benefit and disadvantages of fecal bacteria transplantation. FMT was performed after steroid-refractory GI-GvHD was diagnosed. All the patients were given second-line immunosuppressant treatment. Only the FMT group received FMT. The Center for International Blood and Marrow Transplant Research (CIBMTR) criteria were used to assess the grades of GI GvHD. Criteria for diagnosing steroid-refractory gut GvHD had been described previously. We excluded the patients with uncontrollable infection, irreversible organ failure, and other abnormal conditions that might interfere with the evaluation(Supplement Protocol).

The study was registered with ClinicalTrials.gov as FMT2017002 (#NCT03148743).

**Procedures**

The fecal materials were handled in sealed, fully automatic machines GenFMTer (Nanjing, China). The fecal microbiota collected from four healthy donors (two females aged 23 years, and two males aged 20 years) were conserved in -80℃ with glycerine(Supplement protocol). As these patients couldn't tolerate gastroscopy or enteroscopy, forty to fifty ml of frozen fecal microbiota were suspended in 150-200 ml of warm normal saline and delivered into the intestine of the recipients through a nasojejunal tube or gastric tube after steroid-refractory GI-GvHD was diagnosed. If not got improvement, FMT would be repeated in the following week.

**Outcomes**

The primary outcomes were described with EFS and OS at Day 90 after steroid-refractory GI-GvHD was diagnosed; EFS and OS after steroid-refractory GI-GvHD was recorded till the end of November1 2018

Secondary outcomes were: clinical remission or partial remission at Day 14th, Day 21th and Day 28th after steroid-refractory GI-GvHD was diagnosed.

FMT efficacy were evaluated according to the severity of symptoms such as abdominal pain, diarrhea (frequency and volume), and bloody purulent stool within 14 and 21 days after FMT was accomplished. For abdominal pain score, 0.5 was given to occasional pain, 1 to mild pain, 2 to moderate pain, 3 to severe pain without intervention, and 4 to severe pain with intervention. Clinical remission was defined as a condition in which diarrhea and intestinal spasms and/or bleeding disappeared, or stool volume decreased by≥500mL on average within 3 days. Clinical improvement was defined as a condition in which the stool volume decreased by <500mL, or the abdominal pain value and bleeding relieved. The period during follow-up after first FMT with no progress of GI-GvHD, no death, no GvHD involvement in other organs, no new infection with CMV and EBV were deﬁned as event free survival time (EFS). OS (overall survival) referred to the period from when steroid-refractory GI-GvHD was diagnosed to November1 2018. All deaths, including relapse related or other causes in these period, were included in the statistics.

For each patient, the safety was evaluated according to adverse events (including death or drop-out) during FMT and follow-up time.

**DNA extraction**

Fecal samples were stored at −80° C until DNA extraction. DNA was extracted from 200 mg samples using a DNA extration kit ( QIAamp DNA Stool Mini Kit;QIAGEN, Hilden, Germany), following the manufacturer’s instructions after preparation. DNA concentration and purity was checked by running the samples on 1.2% agarose gels.

**PCR amplification of 16SrDNA genes and Miseq sequencing**

Polymerase chain reaction (PCR) amplification of target gene was performed using general primers 16S V4-V5: 515F 5'-GTGCCAGCMGCCGCGGTAA-3' ; 926R 5'-CCGTCAATTCMTTTGAGTTT-3'. The primers also contained the Illumina 5'overhang adapter sequences for two-step amplicon library building, following manufacturer's instructions for the overhang sequences. The initial PCR reactions were carried out in 50 μL reaction volumes with 1-2 μL DNA template, 200 uM dNTPs, 0.2 uM of each primer, 5X reaction buffer 10uL and 1U Phusion DNA Polymerase (New England Biolabs, USA). PCR conditions consisted of initial denaturation at 94 °C for 2 min, followed by 25 cycles of denaturation at 94 °C for 30 s, annealing at 56 C for 30 s and extension at 72 °C for 30 s, with a final extension of 72 °C for 5 min. The second step PCR with dual 8-base barcodes was used for multiplexing. Eight cycle PCR reactions were used to incorporate two unique barcodes to either end of the 16S amplicons. Cycling conditions consisted of one cycle of 94 °C for 3 min, followed by eight cycles of 94 °C for 30 s, 56 °C for 30 s and 72 °C for 30 s, followed by a final extension cycle of 72 °C for 5 min. Prior to library pooling, the barcoded PCR products were purified using a DNA gel extraction kit (Axygen, USA) and quantified using the FTC -3000 TM real-time PCR(Funglyn Shanghai). The PCR products from different samples were indexed and mixed at equal ratios for sequencing by 2*300bp paired-end sequencing on the MiSeq platform using MiSeq v3 Reagent Kit (Illumina) at TinyGen Bio-Tech Co., Ltd. (Shanghai,China)

**Bioinformatic analysis**

The raw fastq files were demultiplexed based on the barcode. PE reads for all samples were run through Trimmomatic (version 0.35) to remove low quality base pairs using these parameters (SLIDINGWINDOW: 50:20 MINLEN: 50). Trimmed reads were then further merged using FLASH program (version 1.2.11) with default parameters. The low quality contigs were removed based on screen.seqs command using the following filtering parameters, maxambig=0, minlength = 200, maxlength =580, maxhomop= 8.

The 16S sequences were analyzed using a combination of software mothur (version 1.33.3), UPARSE (version v8.1.1756), and R (version 3.6.0).

The demultiplexed reads were clustered at 97% sequence identity into operational taxonomic units (OTUs) and the singleton OTUs were deleted using the UPARSE pipeline.

The OTU representative sequences were assignment for taxonomy against Silva 128 database with confidence score ≥ 0.6 by the classify.seqs command in mothur. OTU taxonomies (from Phylum to Species) were determined based on NCBI.

For the alpha-diversity analysis, Shannon, simpson, Chao1, ACE index and rarefaction curves were calculated were using mothur and plotted by R.

For the beta-diversity metrics, the weighted and unweighted UniFrac distance matrix were calculated using mothur and visualized with Principal Coordinate Analysis (PCoA) and tree by R. The bray curits metrics were calculated by R and visualized also by R.

**Statistical Analysis**

SPSS16.0 (SPSS, Inc., Chicago, IL, USA) for statistical analyses was used to construct actuarial rate curves and to calculate log-rank hazard ratios (HRs) and significance determinations, Fisher's exact tests, and risk determinations. Cochran's and mantel-haenszel statistical methods examined the differences between the groups. The survival package from “R” statistical software (Vienna, Austria) was used for permutation tests. For the latter, variables were determined for each day of the patient's hospitalization and HRs, confidence intervals, and significance determinations were calculated using Cox proportional hazards models with time varying covariates.
